# Multivisceral Oncological Resections Involving the Pancreas: Protocol for a Systematic Review and Meta-Analysis

**DOI:** 10.2196/54089

**Published:** 2024-06-11

**Authors:** Melina Neuhaus, Juliane Friedrichs, Maurizio Grilli, Jörg Ukkat, Johannes Klose, Ulrich Ronellenfitsch, Jörg Kleeff, Artur Rebelo

**Affiliations:** 1 Uniklinikum Halle Halle Germany; 2 Professional Life Science Information Service Karlsruhe Germany

**Keywords:** meta-analysis, systematic review, multivisceral resection, pancreatic resection, oncological resection, surgery, outcomes, mortality, morbidity, survival, cancer, tumor, pancreas

## Abstract

**Background:**

With the continuous advancement of cancer treatments, a comprehensive analysis of the impact of multivisceral oncological pancreatic resections on morbidity, mortality, and long-term survival is currently lacking.

**Objective:**

This manuscript presents the protocol for a systematic review and meta-analysis designed to summarize the existing evidence concerning the outcomes of multivisceral oncological pancreatic resections across diverse tumor entities.

**Methods:**

We will conduct a systematic search of the PubMed or MEDLINE, Embase, Cochrane Library, CINAHL, and ClinicalTrials.gov databases in strict accordance with the PRISMA (Preferred Reporting Items for Systematic Reviews and Meta-Analyses) guidelines. The predefined outcomes encompass postoperative mortality, postoperative morbidity, overall and disease-free survival (1- to 5-year survival rates), the proportion of macroscopically complete (R0) resections (according to the Royal College of Pathologists definition), duration of hospital stay (in days), reoperation rate (%), postoperative complications (covering all complications according to the Clavien-Dindo classification), as well as pancreatic fistula, postpancreatectomy hemorrhage, and delayed gastric emptying (all according to the definitions of the International Study Group of Pancreas Surgery).

**Results:**

Systematic database searches will begin in July 2024. The completion of the meta-analysis is anticipated by December 2024. Before completion, the literature search will be checked for new publications that must be considered in the context of the work.

**Conclusions:**

The forthcoming findings will provide an up-to-date overview of the feasibility, safety, and oncological efficacy of multivisceral pancreatic resections across diverse tumor entities. This data will serve as a valuable resource for health care professionals and patients to make well-informed clinical decisions.

**Trial Registration:**

PROSPERO CRD42023437858; https://tinyurl.com/bde5xmfw

**International Registered Report Identifier (IRRID):**

PRR1-10.2196/54089

## Introduction

Complete resection stands as the principal curative recourse for nonmetastatic solid malignancies. However, in instances of locally advanced stages, often involving encroachment of adjacent organs or structures, the mere excision of the tumor’s origin may prove insufficient. In such scenarios, a multivisceral resection is necessary, entailing the removal of proximate organs [1-4].

Among abdominal tumors in locally advanced stages such as sarcomas, colon cancer, pancreatic cancer, and gastric cancer, the organs most frequently subjected to resection encompass the colon, gallbladder, stomach, liver, kidney, and notably, the pancreas [1,3,5,6]. While isolated pancreatic operations are acknowledged as intricate interventions bearing considerable risks, including considerable mortality and morbidity rates [2,4], a noteworthy proportion of patients, roughly one-third, undergo pancreatic resection as part of a multivisceral resection [7]. If oncological multivisceral resections include high risk interventions such as a pancreas resection, this can be associated with an additional increase in complication rates [2,3,5,8].

The adoption of such aggressive resections can potentially enhance the prospects of achieving negative resection margins, longer survival times, and even cure [5,9,10]. However, due to the increased surgical trauma, these interventions also introduce supplementary hazards that can compromise outcomes and diminish survival prospects [2,6,8].

The evidence concerning the impact of multivisceral oncological pancreatic resections on morbidity, mortality, and long-term survival across varied tumor entities is characterized by heterogeneity [5,11]. Consequently, formulating evidence-based decisions becomes a formidable challenge.

We plan to conduct a systematic review with meta-analysis to summarize the currently available evidence on morbidity, mortality, and long-term survival in these extensive interventions.

## Methods

The literature search and data analysis will be conducted in accordance with the PRISMA (Preferred Reporting Items for Systematic Reviews and Meta-Analyses) guidelines [12]. The study has been registered in the PROSPERO (International Prospective Register of Systematic Reviews) database (CRD42023437858) [13].

### Search Strategy

With a predefined search strategy (Multimedia Appendix 1), publications will be identified from the databases PubMed or MEDLINE, Cochrane Library, CINAHL, and ClinicalTrials.gov. The search will be performed on articles that were published between database inception and a defined search date. The search strategies used in the individual databases will be documented. Furthermore, the reference list of the included studies will be manually searched to find relevant articles. Titles and abstracts will be evaluated independently in a standardized manner by 2 authors to assess eligibility for inclusion or exclusion. All the potential studies identified from the search will be coded as either “retrieve” (eligible, potentially eligible, or unclear) or “do not retrieve.” For studies coded “retrieve,” 2 reviewers will independently screen the full text and recommend inclusion or exclusion. Disagreements between reviewers will be resolved by consensus. If no agreement can be reached, a third reviewer will decide whether to include the study.

### Inclusion and Exclusion Criteria

Publications of observational studies and randomized controlled trials of patients undergoing multivisceral pancreatic resection will be considered. The incorporation of both observational studies and randomized controlled trials may introduce heterogeneity in study designs, potentially impacting the overall quality of evidence. Nevertheless, the inclusion of only one of the 2 study designs may result in insufficient statistical power to reliably detect treatment effects. By defining inclusion and exclusion criteria and assessing the risk of bias, we try to ensure the highest quality and reduce variability. Concerning pancreatic malignancies, multivisceral pancreatic resection refers to the excision of organs beyond the pancreas or spleen in cases of distal pancreatectomy. For multivisceral pancreaticoduodenectomies or total pancreatectomies, the resection encompasses additional organs other than the distal two-thirds of the stomach, the duodenum with the first jejunal loop, the bile duct including the gallbladder, and the spleen. It is important to note that additional procedures like portal vein resection or splenectomy are not categorized as multivisceral resections within the respective resection types. In cases involving nonpancreatic malignancies, any surgical procedure that includes resection of the pancreas along with other organs will be classified as a multivisceral resection. Notably, patients who underwent isolated pancreatic resection for pancreatic metastasis or revision pancreatectomies will be excluded from this analysis. Patients who were not undergoing oncological resections (eg, surgery for traumatic lesions) or those who did not undergo surgery with “curative intent” will also be excluded from the study.

**Figure 1:**
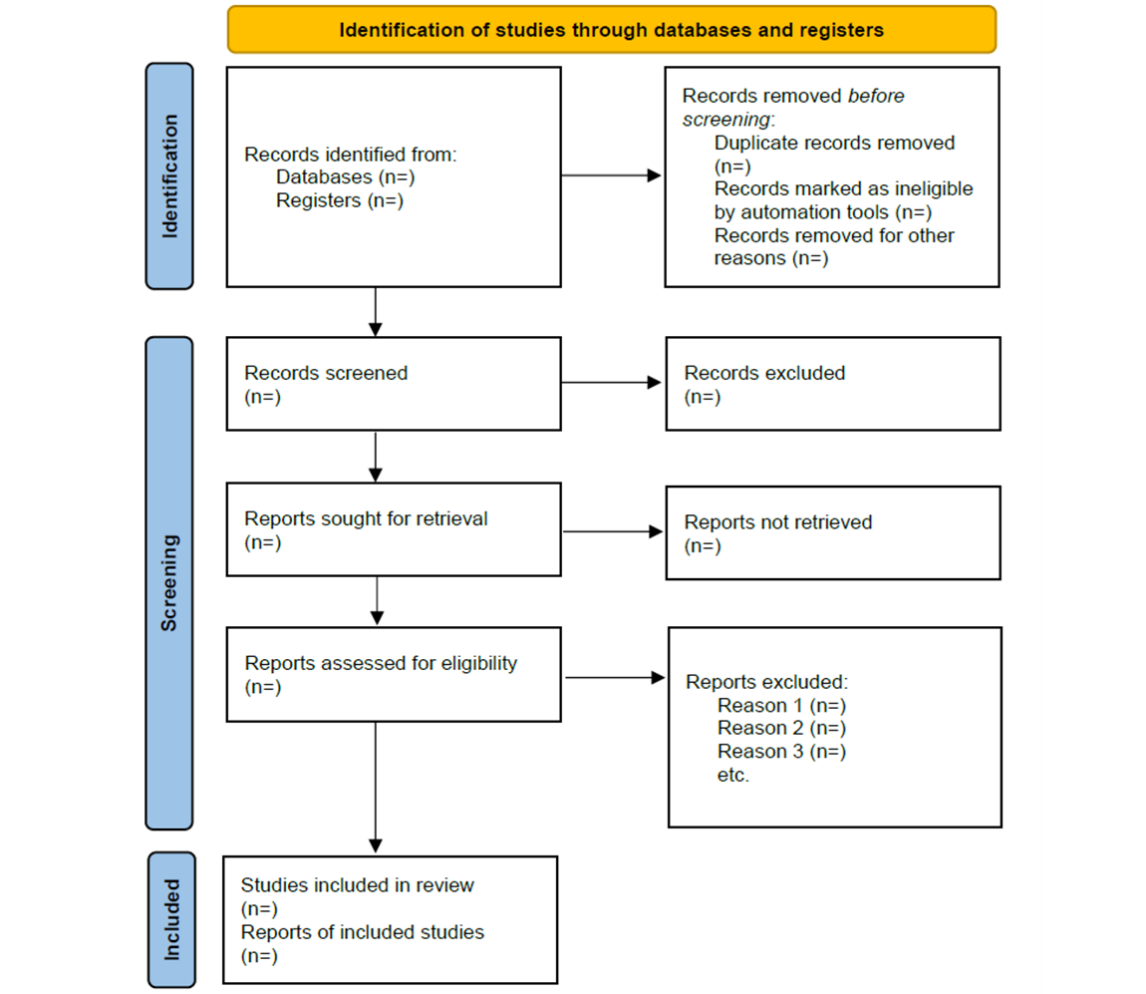
PRISMA (Preferred Reporting Items for Systematic Reviews and Meta-Analyses) 2020 flow diagram.

Reviews, clinical case reports or case series, and other scientific papers reporting on fewer than 10 patients, as well as comments and letters, will not be considered. There are no language restrictions. The details of the study selection process will be summarized in a flowchart (Figure 1).

### Data Collection

Data from the individual included studies will be extracted separately by 2 authors and collected in a dedicated database. The following descriptive data will be documented for each selected study: first author, year of publication, inclusion period of the study, country where the study was conducted, study size, and median follow-up time.

The distribution of the following patient and operation characteristics will be documented: age (in years), sex (male or female), comorbidities (according to the Charlson Comorbidity Index), American Society of Anesthesiologists classification (6 categories), Eastern Cooperative Oncology Group performance status (scale 0 to 5), tumor entity (eg, pancreatic adenocarcinoma, pancreatic neuroendocrine tumors, cystic pancreatic lesions, lymphoma, sarcoma, gastrointestinal stromal tumors, cholangiocarcinoma, other type of carcinoma, and nonpancreatic-neuroendocrine tumors), tumor stage (according to TNM-classification), neoadjuvant, adjuvant or radiotherapy (yes, no, or regimen), type of pancreatic resection (total pancreatectomy, distal pancreatectomy, pancreaticoduodenectomy, or central pancreatectomy), resected organs and structures, date and duration of surgery (in minutes), type of surgical access (open surgery, laparoscopic surgery, or robotic assisted surgery), intraoperative complications (according to Satava’s classification and type), as well as blood loss (in milliliters, method used).

The following predefined outcomes will be extracted: mortality (in-hospital, 30-day, or 90-day), morbidity, overall survival (1- to 5-year survival rates), duration of follow-up, recurrence-free survival, proportion of macroscopically complete resection (%), duration of hospital and intensive care unit stay (days), reoperation rate (%), and postoperative bleeding. Postoperative complications are scored and classified using the Clavien-Dindo classification of surgical complications [14]. When the Clavien-Dindo classification is not available, complications will be categorized as major and minor when possible. Resection margins, including transection and circumferential margins of the pancreas, are categorized according to the definition of the Royal College of Pathologists and classified into R0 (distance margin to tumor ≥1 mm), R1 (distance margin to tumor <1 mm), and R2 (macroscopically positive margin). Complications, readmissions, and mortality are all recorded up to 90 days postoperatively. Pancreatic fistula, postpancreatectomy hemorrhage, and delayed gastric emptying are categorized according to the definitions of the International Study Group of Pancreas Surgery (Multimedia Appendices 1 and 2) [15].

For each study, the risk of bias will be assessed using the ROBINS-I (risk of bias in nonrandomized studies of interventions) tool suggested by the Cochrane collaboration [16]. An ideal randomized controlled trial on the pertinent research question will be conceived and emulated. The actual studies included in the meta-analysis will be compared with this emulated trial regarding their risk of bias in the following domains:

1. Preintervention domains: bias due to confounding and bias in the selection of participants for the study.

2. Intervention domain: bias in the classification of interventions.

3. Postintervention domains: bias due to deviations from intended interventions, bias due to missing data, bias in measurement of the outcome, and bias in selection of the reported result.

For each domain, the tool foresees signaling questions whose response options are yes, probably yes, probably no, no, and no information. Based on the responses, the risk of bias for each domain will be judged as low, moderate, serious, critical, or no information. From the risk of bias for the single domains, an overall risk of bias for the study will be ascertained according to Textbox 1.

For randomized controlled trials, the risk of bias 2 (RoB 2), the Cochrane risk-of-bias tool for randomized trials, will be used [16]. Like the ROBINS-I tool, RoB 2 is structured into domains of bias, with signaling questions for each domain. Based on the responses and the risk of bias for the single domains, an overall risk of bias for the study will be ascertained (Table 1).

**Inclusion and exclusion criteria.**

**Inclusion criteria**

- Article or study type: observational studies and randomized controlled trials of patients undergoing multivisceral pancreatic resections.
- Study population
  Concerning pancreatic malignancies: resection of organs beyond the pancreas or spleen in cases of distal pancreatectomy or resection of additional organs other than the distal two-thirds of the stomach, duodenum with the first jejunal loop, bile duct including the gallbladder, and spleen for multivisceral pancreaticoduodenectomies or total pancreatectomies.
  Concerning nonpancreatic malignancies: any surgical procedure that includes resection of the pancreas along with other organs will be classified as a multivisceral resection.
- Reported outcomes: at least one of the following:
  Mortality (in-hospital, 30-day, or 90-day)
  Morbidity
  Long-term survival (1-5–year survival)
  date of last follow-up and status
  Recurrence-free survival
  Proportion of macroscopically complete resection
  Resection margins, including pancreatic transection and circumferential margins
  Duration of hospital or intensive care unit stay
  Reoperation rate
  Postoperative complications (eg, pancreatic fistula, delayed gastric emptying, or postpancreatectomy hemorrhage)
- Language: all languages.

**Exclusion criteria**

- Article or study type:
  Reviews
  Case reports
  Case series with fewer than 10 patients
  Commentaries
  Letters
- Study population:
  Additional procedures like portal vein resection or splenectomy are not categorized as multivisceral resections in the case of distal pancreatectomy, multivisceral pancreaticoduodenectomies, or total pancreatectomies.
  Patients who underwent isolated pancreatic resection for pancreatic metastasis or revision pancreatectomies.
  Patients who were not undergoing oncological resections or did not undergo surgery had “curative intent.”
- Reported outcomes: none of the outcomes mentioned as inclusion criteria.

**Table 1:** Bias judgment [16].

Overall risk of bias judgment	Interpretation	Criteria
Low risk of bias	The study is comparable to a well-performed randomized trial.	The study is judged to be at low risk of bias for all domains for this result.
Moderate risk of bias	The study appears to provide sound evidence for a nonrandomized study but cannot be considered com- parable to a well-performed randomized trial.	The study is judged to be at low or moderate risk of bias for all domains.
Serious risk of bias	The study has one or more important problems.	The study is judged to be at serious risk of bias in at least one domain but not at critical risk of bias in any domain.

### Statistical Analysis

As stated, all studies will undergo a qualitative analysis by examining data through techniques like coding and thematic analysis to uncover patterns and meanings. It aims to provide a rich understanding of the subject by considering context, multiple perspectives, and researcher reflexivity. An analysis will be performed for all comparative trials. A separate analysis will be performed for randomized trials. Furthermore, a meta-analysis will be conducted exclusively for comparative studies, which involve a cohort of patients undergoing nonmultivisceral oncological resections for direct comparison. Subgroup analyses will be executed for each distinct tumor entity encompassed within the study, including sarcoma, colon cancer, pancreatic cancer, gastric cancer, and other oncological conditions. Also, a stratification according to different tumor types, for example, pancreatic ductal adenocarcinoma or pancreatic neuroendocrine tumors, will be conducted.

The Review Manager software (version 5.4; The Cochrane collaboration) will be used. A random effects model will be used to assess the effect estimate. Visualization will be facilitated through forest and funnel plots to illustrate the magnitude of the effect. Dichotomous data will be subjected to odd ratio analysis with 95% CIs. Continuous data will undergo mean difference calculations alongside 95% CIs, and if continuous outcomes are assessed on different scales, a standardized mean difference with corresponding 95% CIs will be determined. When the studies do not report mean difference and standardized mean differences, these will be calculated using the methods described by the guidelines of the Cochrane collaboration [17] and Hozo et al [18]. If hazard ratios are not reported, the team may digitize the curve and calculate them. If the proportional hazards assumption is not met, the team might explore alternative statistical methods such as stratified analysis or time-dependent covariate analysis to appropriately handle the violation. The 95% CI, heterogeneity, and statistical significance will be reported for each outcome. The chi-square and Kruskal-Wallis tests will be used for the evaluation of statistical significance. A value of *P*<.05 will be considered statistically significant. The outcome “postoperative complications” will be assessed when possible, according to the Clavien-Dindo classification [14].

Sensitivity analyses will be conducted according to the risk of bias ascertained as previously described. For these, all studies with a high or serious risk of bias will be excluded, and the analyses of the outcomes, as previously described, will be conducted. To determine the quality of the evidence, the GRADE (Grading of Recommendations Assessment, Development, and Evaluation) criteria (study limitations, consistency of effect, imprecision, indirectness, and publication bias) will be used. In accordance with GRADE, evidence will be distinguished between high, moderate, low, or very low [19].

### Ethical Considerations

Due to the nature of the data used in this meta-analysis, which involves aggregate information from previously published studies, ethical approval is deemed unnecessary.

## Results

Database searches will commence in July 2024. The meta-analysis will be completed by December 2024. Before completion, the literature search is checked for new publications that must be taken into account in the context of the work.

## Discussion

This systematic review with meta-analysis will synthesize all available evidence on the feasibility, safety, and oncological effectiveness of multivisceral pancreatic resections in various tumor diseases. Due to the limited number of eligible studies, we include both observational studies and randomized controlled trials, which may introduce heterogeneity in the study designs, potentially affecting the overall quality of the evidence. Nevertheless, the inclusion of only one of the 2 study designs may result in insufficient statistical power to reliably detect treatment effects. Additionally, inherent limitations in retrospective studies might impact the overall quality of the evidence. Hence, we enhance the statistical power by consolidating the findings from both study methods. Through the establishment of clear inclusion and exclusion criteria and rigorous assessment of bias risks, our aim is to uphold the highest quality standards and reduce variability. Furthermore, minor differences in skill levels and learning curves among surgeons, as well as the focus on specific academic research institutions, may impact the generalizability of the findings. We will thoroughly address these limitations in the discussion section and, if feasible, conduct subgroup analyses to enhance the quality of our analysis. This systematic review and meta-analysis will be conducted according to the defined protocol presented here and will be reported following the recommendations stipulated in the PRISMA [12,20] statement, thus ensuring the highest quality standards and minimizing the risk of possible bias. The expected results will provide new information on the prognostic value of multivisceral pancreatic resections in various tumor diseases and thereby support health care professionals and patients in their decision-making.

## Appendix

Multimedia Appendix 1 Search strategy.

Multimedia Appendix 2 Variables.

Multimedia Appendix 3 Index, scores, and definitions.

Multimedia Appendix 4 PRISMA-P (Preferred Reporting Items for Systematic review and Meta-Analysis Protocols) checklist.

## Abbreviations

GRADE: Grading of Recommendations Assessment, Development, and Evaluation
PRISMA: Preferred Reporting Items for Systematic Reviews and Meta-Analyses
PROSPERO: International Prospective Register of Systematic Reviews
RoB 2: risk of bias 2
ROBINS-I: risk of bias in nonrandomized studies of interventions
