# Supplementary material for: Multivisceral Oncological Resections Involving the Pancreas: Protocol for a Systematic Review and Meta-Analysis
Source: JMIR Res Protoc. 2024 Jun 11;13:e54089. doi: 10.2196/54089 (PMC11200041; doi:10.2196/54089)
Supplement: Multimedia Appendix 1 [file resprot_v13i1e54089_app1.docx]

## Search guide overview

| 1 | P |  |
| --- | --- | --- |
| 2 | I |  |
| 3 | 1 AND 2 |  |

## Databases and platforms involved

- PubMed (via NCBI)
- EMBASE (via Elsevier)
- Cochrane Library (via Wiley)
- Cinahl (via Ebsco host)
- ClinicalTrials.Gov (via [www.clinicaltrials.gov](http://www.clinicaltrials.gov))
- ICTRP (via <https://trialsearch.who.int/Default.aspx>)

## Results report

The results were saved and deduplicated in Endnote. For this, the settings DOI/ Author, Year, Title, Secondary Title/ Author, Year, Title, Pages/ Title/ Author, Year were applied. However, some items may appear more than once.

The hits are sorted by database in Endnote. The PubMed hits were the first to be exported to Endnote. These are therefore preferred for deduplication. This means that in the case of duplicates, entries from other databases are removed first. The hits in the trash folder are the removed duplicates.

The hit count for each database in this report relates to the status before deduplication in EndNote.

## PubMed

### P

|  | **"Pancreas"[Majr] OR**  **"Colon"[Majr] OR**  **"Stomach"[Majr] OR**  **"Adrenal Glands"[Majr] OR**  **"Liver"[Majr] OR**  **"Kidney"[Majr] OR**  **"Intestine, Small"[Majr] OR**  **"Spleen"[Majr] OR**  Pancrea*[tiab] OR  colon*[tiab] OR  stomach*[tiab] OR  "Adrenal Gland*"[tiab] OR  liver*[tiab] OR  kidney*[tiab] OR  "small intestine*"[tiab] OR  spleen*[tiab] |  |
| --- | --- | --- |

### I

|  | Multivisceral*[tiab] OR  "Added visceral"[tiab:~1] OR  "Additional visceral"[tiab:~1] OR  "broad range visceral"[tiab:~1] OR  "Diverse visceral"[tiab:~1] OR  "Extended visceral"[tiab:~1] OR  "Extra visceral"[tiab:~1] OR  "Further visceral"[tiab:~1] OR  "Incremental visceral"[tiab:~1] OR  "Manifold visceral"[tiab:~1] OR  "Many visceral"[tiab:~1] OR  "More visceral"[tiab:~1] OR  "Multi visceral"[tiab:~1] OR  "Numerous visceral"[tiab:~1] OR  "Plural visceral"[tiab:~1] OR  "Plus visceral"[tiab:~1] OR  "Poly visceral"[tiab:~1] OR  "Several visceral"[tiab:~1] OR  "Supplement visceral"[tiab:~1] OR  "variety visceral"[tiab:~1] OR  "Various visceral"[tiab:~1] OR  "wide array visceral"[tiab:~1] OR |  |
| --- | --- | --- |
|  | "Added resection"[tiab:~1] OR  "Additional resection"[tiab:~1] OR  "broad range resection"[tiab:~1] OR  "Diverse resection"[tiab:~1] OR  "Extended resection"[tiab:~1] OR  "Extra resection"[tiab:~1] OR  "Further resection"[tiab:~1] OR  "Incremental resection"[tiab:~1] OR  "Manifold resection"[tiab:~1] OR  "Many resection"[tiab:~1] OR  "More resection"[tiab:~1] OR  "Multi resection"[tiab:~1] OR  "Numerous resection"[tiab:~1] OR  "Plural resection"[tiab:~1] OR  "Plus resection"[tiab:~1] OR  "Poly resection"[tiab:~1] OR  "Several resection"[tiab:~1] OR  "Supplement resection"[tiab:~1] OR  "variety resection"[tiab:~1] OR  "Various resection"[tiab:~1] OR  "wide array resection"[tiab:~1] |  |

### Strings

| 1 | "Pancreas"[MeSH Major Topic] OR "Colon"[MeSH Major Topic] OR "Stomach"[MeSH Major Topic] OR "Adrenal Glands"[MeSH Major Topic] OR "Liver"[MeSH Major Topic] OR "Kidney"[MeSH Major Topic] OR "intestine, small"[MeSH Major Topic] OR "Spleen"[MeSH Major Topic] OR "pancrea*"[Title/Abstract] OR "colon*"[Title/Abstract] OR "stomach*"[Title/Abstract] OR "adrenal gland*"[Title/Abstract] OR "liver*"[Title/Abstract] OR "kidney*"[Title/Abstract] OR "small intestine*"[Title/Abstract] OR "spleen*"[Title/Abstract] | 2805175 |
| --- | --- | --- |
| 2 | "multivisceral*"[Title/Abstract] OR "Added visceral"[Title/Abstract:~1] OR "Additional visceral"[Title/Abstract:~1] OR "broad range visceral"[Title/Abstract:~1] OR "Diverse visceral"[Title/Abstract:~1] OR "Extended visceral"[Title/Abstract:~1] OR "Extra visceral"[Title/Abstract:~1] OR "Further visceral"[Title/Abstract:~1] OR "Incremental visceral"[Title/Abstract:~1] OR "Manifold visceral"[Title/Abstract:~1] OR "Many visceral"[Title/Abstract:~1] OR "More visceral"[Title/Abstract:~1] OR "Multi visceral"[Title/Abstract:~1] OR "Numerous visceral"[Title/Abstract:~1] OR "Plural visceral"[Title/Abstract:~1] OR "Plus visceral"[Title/Abstract:~1] OR "Poly visceral"[Title/Abstract:~1] OR "Several visceral"[Title/Abstract:~1] OR "Supplement visceral"[Title/Abstract:~1] OR "variety visceral"[Title/Abstract:~1] OR "Various visceral"[Title/Abstract:~1] OR "wide array visceral"[Title/Abstract:~1] OR "Added resection"[Title/Abstract:~1] OR "Additional resection"[Title/Abstract:~1] OR "broad range resection"[Title/Abstract:~1] OR "Diverse resection"[Title/Abstract:~1] OR "Extended resection"[Title/Abstract:~1] OR "Extra resection"[Title/Abstract:~1] OR "Further resection"[Title/Abstract:~1] OR "Incremental resection"[Title/Abstract:~1] OR "Manifold resection"[Title/Abstract:~1] OR "Many resection"[Title/Abstract:~1] OR "More resection"[Title/Abstract:~1] OR "Multi resection"[Title/Abstract:~1] OR "Numerous resection"[Title/Abstract:~1] OR "Plural resection"[Title/Abstract:~1] OR "Plus resection"[Title/Abstract:~1] OR "Poly resection"[Title/Abstract:~1] OR "Several resection"[Title/Abstract:~1] OR "Supplement resection"[Title/Abstract:~1] OR "variety resection"[Title/Abstract:~1] OR "Various resection"[Title/Abstract:~1] OR "wide array resection"[Title/Abstract:~1] | 10537 |
| 3 | #1 AND #2 | 3737 |

## Embase

### P

|  | **'pancreas'/exp/mj OR**  **'colon'/exp/mj OR**  **'stomach'/exp/mj OR**  **'adrenal gland'/exp/mj OR**  **'liver'/exp/mj OR**  **'kidney'/exp/mj OR**  **'small intestine'/exp/mj OR**  **'spleen'/exp/mj OR**  Pancrea*:ti,ab OR  colon*:ti,ab OR  stomach*:ti,ab OR  "Adrenal Gland*":ti,ab OR  liver*:ti,ab OR  kidney*:ti,ab OR  "small intestine*":ti,ab OR  spleen*:ti,ab |  |
| --- | --- | --- |

### I

|  | Multivisceral*:ti,ab OR  ((Added NEAR/2 visceral) OR  (Additional NEAR/2 visceral) OR  (broad NEAR/2 range NEAR/2 visceral) OR  (Diverse NEAR/2 visceral) OR  (Extended NEAR/2 visceral) OR  (Extra NEAR/2 visceral) OR  (Further NEAR/2 visceral) OR  (Incremental NEAR/2 visceral) OR  (Manifold NEAR/2 visceral) OR  (Many NEAR/2 visceral) OR  (More NEAR/2 visceral) OR  (Multi NEAR/2 visceral) OR  (Numerous NEAR/2 visceral) OR  (Plural NEAR/2 visceral) OR  (Plus NEAR/2 visceral) OR  (Poly NEAR/2 visceral) OR  (Several NEAR/2 visceral) OR  (Supplement NEAR/2 visceral) OR  (variety NEAR/2 visceral) OR  (Various NEAR/2 visceral) OR  (wide array NEAR/2 visceral)):ti,ab OR |  |
| --- | --- | --- |
|  | ((Added NEAR/2 resection) OR  (Additional NEAR/2 resection) OR  (broad NEAR/2 range NEAR/2 resection) OR  (Diverse NEAR/2 resection) OR  (Extended NEAR/2 resection) OR  (Extra NEAR/2 resection) OR  (Further NEAR/2 resection) OR  (Incremental NEAR/2 resection) OR  (Manifold NEAR/2 resection) OR  (Many NEAR/2 resection) OR  (More NEAR/2 resection) OR  (Multi NEAR/2 resection) OR  (Numerous NEAR/2 resection) OR  (Plural NEAR/2 resection) OR  (Plus NEAR/2 resection) OR  (Poly NEAR/2 resection) OR  (Several NEAR/2 resection) OR  (Supplement NEAR/2 resection) OR  (variety NEAR/2 resection) OR  (Various NEAR/2 resection) OR  (wide array NEAR/2 resection)):ti,ab |  |

### Strings

| 1 | 'pancreas'/exp/mj OR 'colon'/exp/mj OR 'stomach'/exp/mj OR 'adrenal gland'/exp/mj OR 'liver'/exp/mj OR 'kidney'/exp/mj OR 'small intestine'/exp/mj OR 'spleen'/exp/mj OR pancrea*:ti,ab OR colon*:ti,ab OR stomach*:ti,ab OR 'adrenal gland*':ti,ab OR liver*:ti,ab OR kidney*:ti,ab OR 'small intestine*':ti,ab OR spleen*:ti,ab | 3646042 |
| --- | --- | --- |
| 2 | multivisceral*:ti,ab,kw OR ((added NEAR/4 visceral):ti,ab,kw) OR ((additional NEAR/4 visceral):ti,ab,kw) OR ((broad NEAR/4 range NEAR/4 visceral):ti,ab,kw) OR ((diverse NEAR/4 visceral):ti,ab,kw) OR ((extended NEAR/4 visceral):ti,ab,kw) OR ((extra NEAR/4 visceral):ti,ab,kw) OR ((further NEAR/4 visceral):ti,ab,kw) OR ((incremental NEAR/4 visceral):ti,ab,kw) OR ((manifold NEAR/4 visceral):ti,ab,kw) OR ((many NEAR/4 visceral):ti,ab,kw) OR ((more NEAR/4 visceral):ti,ab,kw) OR ((multi NEAR/4 visceral):ti,ab,kw) OR ((numerous NEAR/4 visceral):ti,ab,kw) OR ((plural NEAR/4 visceral):ti,ab,kw) OR ((plus NEAR/4 visceral):ti,ab,kw) OR ((poly NEAR/4 visceral):ti,ab,kw) OR ((several NEAR/4 visceral):ti,ab,kw) OR ((supplement NEAR/4 visceral):ti,ab,kw) OR ((variety NEAR/4 visceral):ti,ab,kw) OR ((various NEAR/4 visceral):ti,ab,kw) OR (wide:ti,ab,kw AND ((array NEAR/4 visceral):ti,ab,kw)) | 16996 |
| 3 | #1 AND #2 | 6560 |

**To switch off PubMed**

| 4 | #3 NOT ([medline]/lim OR [pubmed-not-medline]/lim) | 3581 |
| --- | --- | --- |

**To exclude document types not of interest**

| 5 | #4 NOT ('Conference Abstract'/it OR 'Note'/it) | 659 |
| --- | --- | --- |

## Cochrane Library

### P

|  | **[mh "Pancreas"] OR**  **[mh "Colon"] OR**  **[mh "Stomach"] OR**  **[mh "Adrenal Glands"] OR**  **[mh "Liver"] OR**  **[mh "Kidney"] OR**  **[mh "Intestine, Small"] OR**  **[mh "Spleen"] OR**  Pancrea*:ti,ab OR  colon*:ti,ab OR  stomach*:ti,ab OR  Adrenal NEAR/1 Gland*:ti,ab OR  liver*:ti,ab OR  kidney*:ti,ab OR  small NEAR/1 intestine*:ti,ab OR  spleen*:ti,ab | 144861 |
| --- | --- | --- |

### I

|  | (Multivisceral* OR  (Added NEAR/1 visceral) OR  (Additional NEAR/1 visceral) OR  (broad NEAR/1 range NEAR/1 visceral) OR  (Diverse NEAR/1 visceral) OR  (Extended NEAR/1 visceral) OR  (Extra NEAR/1 visceral) OR  (Further NEAR/1 visceral) OR  (Incremental NEAR/1 visceral) OR  (Manifold NEAR/1 visceral) OR  (Many NEAR/1 visceral) OR  (More NEAR/1 visceral) OR  (Multi NEAR/1 visceral) OR  (Numerous NEAR/1 visceral) OR  (Plural NEAR/1 visceral) OR  (Plus NEAR/1 visceral) OR  (Poly NEAR/1 visceral) OR  (Several NEAR/1 visceral) OR  (Supplement NEAR/1 visceral) OR  (variety NEAR/1 visceral) OR  (Various NEAR/1 visceral) OR  (wide NEAR/1 array NEAR/1 visceral)):ti,ab OR  (Added NEAR/1 resection) OR  (Additional NEAR/1 resection) OR  (broad NEAR/1 range NEAR/1 resection) OR  (Diverse NEAR/1 resection) OR  (Extended NEAR/1 resection) OR  (Extra NEAR/1 resection) OR  (Further NEAR/1 resection) OR  (Incremental NEAR/1 resection) OR  (Manifold NEAR/1 resection) OR  (Many NEAR/1 resection) OR  (More NEAR/1 resection) OR  (Multi NEAR/1 resection) OR  (Numerous NEAR/1 resection) OR  (Plural NEAR/1 resection) OR  (Plus NEAR/1 resection) OR  (Poly NEAR/1 resection) OR  (Several NEAR/1 resection) OR  (Supplement NEAR/1 resection) OR  (variety NEAR/1 resection) OR  (Various NEAR/1 resection) OR  (wide NEAR/1 array NEAR/1 resection):ti,ab | 406 |
| --- | --- | --- |

### Strings

1-2 as in the tables above

| 3 | #1 AND #2 | 130 |
| --- | --- | --- |

## CINAHL

### P

|  | **[mh "Pancreas"] OR**  **[mh "Colon"] OR**  **[mh "Stomach"] OR**  **[mh "Adrenal Glands"] OR**  **[mh "Liver"] OR**  **[mh "Kidney"] OR**  **[mh "Intestine, Small"] OR**  **[mh "Spleen"] OR**  (TX (Pancrea* OR  colon* OR  stomach* OR  Adrenal N1 Gland* OR  liver* OR  kidney* OR  small N1 intestine* OR  spleen*)) |  |
| --- | --- | --- |

### I

|  | (TX (Multivisceral* OR  Added N1 visceral OR  Additional N1 visceral OR  broad N1 range N1 visceral OR  Diverse N1 visceral OR  Extended N1 visceral OR  Extra N1 visceral OR  Further N1 visceral OR  Incremental N1 visceral OR  Manifold N1 visceral OR  Many N1 visceral OR  More N1 visceral OR  Multi N1 visceral OR  Numerous N1 visceral OR  Plural N1 visceral OR  Plus N1 visceral OR  Poly N1 visceral OR  Several N1 visceral OR  Supplement N1 visceral OR  variety N1 visceral OR  Various N1 visceral OR  wide N1 array N1 visceral))  OR  (TX (Added N1 resection OR  Additional N1 resection OR  broad N1 range N1 resection OR  Diverse N1 resection OR  Extended N1 resection OR  Extra N1 resection OR  Further N1 resection OR  Incremental N1 resection OR  Manifold N1 resection OR  Many N1 resection OR  More N1 resection OR  Multi N1 resection OR  Numerous N1 resection OR  Plural N1 resection OR  Plus N1 resection OR  Poly N1 resection OR  Several N1 resection OR  Supplement N1 resection OR  variety N1 resection OR  Various N1 resection OR  wide N1 array N1 resection)) |  |
| --- | --- | --- |

### Strings

| 1 | [mh "Pancreas"] OR [mh "Colon"] OR [mh "Stomach"] OR [mh "Adrenal Glands"] OR [mh "Liver"] OR [mh "Kidney"] OR [mh "Intestine, Small"] OR [mh "Spleen"] OR (TX (Pancrea* OR colon* OR stomach* OR Adrenal N1 Gland* OR liver* OR kidney* OR small N1 intestine* OR spleen*)) | 430679 |
| --- | --- | --- |
| 2 | (TX (Multivisceral* OR Added N1 visceral OR Additional N1 visceral OR broad N1 range N1 visceral OR Diverse N1 visceral OR Extended N1 visceral OR Extra N1 visceral OR Further N1 visceral OR Incremental N1 visceral OR Manifold N1 visceral OR Many N1 visceral OR More N1 visceral OR Multi N1 visceral OR Numerous N1 visceral OR Plural N1 visceral OR Plus N1 visceral OR Poly N1 visceral OR Several N1 visceral OR Supplement N1 visceral OR variety N1 visceral OR Various N1 visceral OR wide N1 array N1 visceral)) OR (TX (Added N1 resection OR Additional N1 resection OR broad N1 range N1 resection OR Diverse N1 resection OR Extended N1 resection OR Extra N1 resection OR Further N1 resection OR Incremental N1 resection OR Manifold N1 resection OR Many N1 resection OR More N1 resection OR Multi N1 resection OR Numerous N1 resection OR Plural N1 resection OR Plus N1 resection OR Poly N1 resection OR Several N1 resection OR Supplement N1 resection OR variety N1 resection OR Various N1 resection OR wide N1 array N1 resection)) | 1597 |
| 3 | S1 AND S2 | 628 |

## ClinicalTrial.gov

<http://www.clinicaltrials.gov/>

### P

|  | Pancreas OR  colon OR  stomach OR  "Adrenal Gland" OR  liver OR  kidney OR  "small intestine" OR  spleen* |  |
| --- | --- | --- |

### I

|  | Multivisceral OR  "Added visceral" OR  "Additional visceral" OR  "broad range visceral" OR  "Diverse visceral" OR  "Extended visceral" OR  "Extra visceral" OR  "Further visceral" OR  "Incremental visceral" OR  "Manifold visceral" OR  "Many visceral" OR  "More visceral" OR  "Multi visceral" OR  "Numerous visceral" OR  "Plural visceral" OR  "Plus visceral" OR  "Poly visceral" OR  "Several visceral" OR  "Supplement visceral" OR  "variety visceral" OR  "Various visceral" OR  "wide array visceral" OR  "Added resection" OR  "Additional resection" OR  "broad range resection" OR  "Diverse resection" OR  "Extended resection" OR  "Extra resection" OR  "Further resection" OR  "Incremental resection" OR  "Manifold resection" OR  "Many resection" OR  "More resection" OR  "Multi resection" OR  "Numerous resection" OR  "Plural resection" OR  "Plus resection" OR  "Poly resection" OR  "Several resection" OR  "Supplement resection" OR  "variety resection" OR  "Various resection" OR  "wide array resection" | ((Added OR  Additional OR  "broad range" OR  Diverse OR  Extended OR  Extra OR  Further OR  Incremental OR  Manifold OR  Many OR  More OR  Multi OR  Numerous OR  Plural OR  Plus OR  Poly OR  Several OR  Supplement OR  variety OR  Various OR  "wide array")  AND  resection) |
| --- | --- | --- |

### Strings

| 1 | (Pancreas OR colon OR stomach OR EXPAND[Concept] "Adrenal Gland" OR liver OR kidney OR EXPAND[Concept] "small intestine" OR spleen*) | 74258 |
| --- | --- | --- |
| 2 | AND  (Multivisceral OR EXPAND[Concept] "Added visceral" OR EXPAND[Concept] "Additional visceral" OR EXPAND[Concept] "broad range visceral" OR EXPAND[Concept] "Diverse visceral" OR EXPAND[Concept] "Extended visceral" OR EXPAND[Concept] "Extra visceral" OR EXPAND[Concept] "Further visceral" OR EXPAND[Concept] "Incremental visceral" OR EXPAND[Concept] "Manifold visceral" OR EXPAND[Concept] "Many visceral" OR EXPAND[Concept] "More visceral" OR EXPAND[Concept] "Multi visceral" OR EXPAND[Concept] "Numerous visceral" OR EXPAND[Concept] "Plural visceral" OR EXPAND[Concept] "Plus visceral" OR EXPAND[Concept] "Poly visceral" OR EXPAND[Concept] "Several visceral" OR EXPAND[Concept] "Supplement visceral" OR EXPAND[Concept] "variety visceral" OR EXPAND[Concept] "Various visceral" OR EXPAND[Concept] "wide array visceral" OR EXPAND[Concept] "Added resection" OR EXPAND[Concept] "Additional resection" OR EXPAND[Concept] "broad range resection" OR EXPAND[Concept] "Diverse resection" OR EXPAND[Concept] "Extended resection" OR EXPAND[Concept] "Extra resection" OR EXPAND[Concept] "Further resection" OR EXPAND[Concept] "Incremental resection" OR EXPAND[Concept] "Manifold resection" OR EXPAND[Concept] "Many resection" OR EXPAND[Concept] "More resection" OR EXPAND[Concept] "Multi resection" OR EXPAND[Concept] "Numerous resection" OR EXPAND[Concept] "Plural resection" OR EXPAND[Concept] "Plus resection" OR EXPAND[Concept] "Poly resection" OR EXPAND[Concept] "Several resection" OR EXPAND[Concept] "Supplement resection" OR EXPAND[Concept] "variety resection" OR EXPAND[Concept] "Various resection" OR EXPAND[Concept] "wide array resection") | 83 |

| 3 | 1 And 2 | 46 |
| --- | --- | --- |

## International Clinical Trials Registry Platform ICTRP (WHO Trials)

<https://trialsearch.who.int/> (simple)

<https://trialsearch.who.int/AdvSearch.aspx> (advanced)

### P

|  | Pancreas OR  colon OR  stomach OR  Adrenal Gland OR  liver OR  kidney OR  small intestine OR  spleen* |  |
| --- | --- | --- |

### I

|  | Multivisceral OR  Added visceral OR  Additional visceral OR  broad range visceral OR  Diverse visceral OR  Extended visceral OR  Extra visceral OR  Further visceral OR  Incremental visceral OR  Manifold visceral OR  Many visceral OR  More visceral OR  Multi visceral OR  Numerous visceral OR  Plural visceral OR  Plus visceral OR  Poly visceral OR  Several visceral OR  Supplement visceral OR  variety visceral OR  Various visceral OR  wide array visceral OR  Added resection OR  Additional resection OR  broad range resection OR  Diverse resection OR  Extended resection OR  Extra resection OR  Further resection OR  Incremental resection OR  Manifold resection OR  Many resection OR  More resection OR  Multi resection OR  Numerous resection OR  Plural resection OR  Plus resection OR  Poly resection OR  Several resection OR  Supplement resection OR  variety resection OR  Various resection OR  wide array resection |  |
| --- | --- | --- |

### Strings (in advanced mode)

| **Fields** | **String** | **Hits** |
| --- | --- | --- |
| 1 (Condition) | Pancreas OR colon OR stomach OR Adrenal Gland OR liver OR kidney OR small intestine OR spleen* | 25494 |
| 2 (Intervention) | Multivisceral OR Added visceral OR Additional visceral OR broad range visceral OR Diverse visceral OR Extended visceral OR Extra visceral OR Further visceral OR Incremental visceral OR Manifold visceral OR Many visceral OR More visceral OR Multi visceral OR Numerous visceral OR Plural visceral OR Plus visceral OR Poly visceral OR Several visceral OR Supplement visceral OR variety visceral OR Various visceral OR wide array visceral OR Added resection OR Additional resection OR broad range resection OR Diverse resection OR Extended resection OR Extra resection OR Further resection OR Incremental resection OR Manifold resection OR Many resection OR More resection OR Multi resection OR Numerous resection OR Plural resection OR Plus resection OR Poly resection OR Several resection OR Supplement resection OR variety resection OR Various resection OR wide array resection | 278 |

| 3 | 1 AND 2 | 63 |
| --- | --- | --- |
