# Supplementary material for: Multivisceral Oncological Resections Involving the Pancreas: Protocol for a Systematic Review and Meta-Analysis
Source: JMIR Res Protoc. 2024 Jun 11;13:e54089. doi: 10.2196/54089 (PMC11200041; doi:10.2196/54089)
Supplement: Multimedia Appendix 2 [file resprot_v13i1e54089_app2.docx]

**Variables**

Patient and Operation Characteristics:

1. Age (in years)

2. Gender (male/female)

3. Charlson Comorbidity Index for summarizing patients' comorbidities

4. American Society of Anesthesiologists (ASA) classification (five categories)

5. Eastern Cooperative Oncology Group (ECOG) performance status (scale zero to four)

6. Tumor entity (pancreatic adenocarcinoma, pancreatic neuroendocrine tumors (NETs), Cystic Pancreatic Lesions, Lymphoma, Sarcoma, gastrointestinal stromal tumors (GISTs), Cholangiocarcinoma, other type of carcinoma, non-pancreatic-NET)

7. Tumor stage (according to TNM classification)

8. Neoadjuvant (yes/no/regimen) or/and adjuvant (yes/no/regimen), chemotherapy, or/and neoadjuvant radiotherapy (yes/no/regimen) or/and adjuvant radiotherapy (yes/no/regimen). Regimen: alkylating agents, antimetabolites, antimicrotubule agents, topoisomerase inhibitors, cytotoxic antibiotics, hormone therapy, targeted therapies, immunotherapy, and combination chemotherapy.

9. Type of pancreatic resection (total pancreatectomy, distal pancreatectomy, pancreaticoduodenectomy, central pancreatectomy)

10. Number and name of the resected organs and structures (colon, stomach, adrenal gland, liver, kidney, small intestine, spleen)

11. Date of surgery

12. Duration of surgery (in minutes)

13. Type of surgical access (open surgery, laparoscopic surgery, robotic assisted surgery)

14. Intraoperative complications, categorized: according to Satava’s classification (five categories) and type (hemorrhage, organ or tissue injury, anesthesia-related complications, cardiovascular complications, respiratory complications, neurological complications)

15. Blood loss (in milliliters, method used)

Predefined Outcomes:

16. Mortality (90-day)

17. Date of last follow-up and Status (Death yes or no)

18. Recurrence (Date yes/no)

19. Duration of ICU stay (days)

20. Duration of hospital stay (days)

21. Reoperation, type and date

22. Postoperative bleeding (international study group of pancreas surgery (ISGPS) Definition)

23. Postoperative Pancreatic fistula (ISGPS Definition)

24. Postoperative Delayed gastric emptying (ISGPS Definition)

25. Postoperative complications scored and classified using Clavien-Dindo classification

26. Resection margins categorized according to the Royal College of Pathologists definition
