# Supplementary material for: Multivisceral Oncological Resections Involving the Pancreas: Protocol for a Systematic Review and Meta-Analysis
Source: JMIR Res Protoc. 2024 Jun 11;13:e54089. doi: 10.2196/54089 (PMC11200041; doi:10.2196/54089)
Supplement: Multimedia Appendix 3 [file resprot_v13i1e54089_app3.docx]

**Index, Scores, and Definitions**

1. Charlson Comorbidity Index: A scoring system used to quantify the burden of comorbidities in patients based on the presence of various medical conditions. Each condition is assigned a weight, and the sum of weights provides an overall comorbidity score. This index helps predict the risk of mortality associated with multiple health conditions.

*Charlson, M. E., Pompei, P., Ales, K. L., & MacKenzie, C. R. (1987). A new method of classifying prognostic comorbidity in longitudinal studies: Development and validation. Journal of Chronic Diseases, 40(5), 373-383.*

- No comorbidity: 0

- Myocardial infarction: 1

- Congestive heart failure: 1

- Peripheral vascular disease: 1

- Cerebrovascular disease: 1

- Dementia: 1

- Chronic pulmonary disease: 1

- Rheumatic disease: 1

- Peptic ulcer disease: 1

- Mild liver disease: 1

- Diabetes without complications: 1

- Diabetes with complications: 2

- Hemiplegia or paraplegia: 2

- Renal disease: 2

- Any malignancy: 2

- Moderate or severe liver disease: 3

- Metastatic solid tumor: 6

- AIDS/HIV: 6

- Score ranges from 0 to 37 or more, with higher scores indicating greater comorbidity burden.

2. American Society of Anesthesiologists (ASA) Classification: A categorization system used to assess the overall health status of patients before surgery. It consists of six classes ranging from ASA I (healthy patient) to ASA VI (brain-dead patient undergoing organ donation).

*American Society of Anesthesiologists. (2014). ASA physical status classification system. Retrieved from https://www.asahq.org/standards-and-guidelines/asa-physical-status-classification-system*

- ASA I: Normal healthy patient

- ASA II: Mild systemic disease

- ASA III: Severe systemic disease

- ASA IV: Severe systemic disease that is a constant threat to life

- ASA V: Moribund patient not expected to survive without surgery

- ASA VI: Brain-dead patient undergoing organ donation

3. Eastern Cooperative Oncology Group (ECOG) Performance Status: A scale ranging from 0 to 5 that measures the functional status of cancer patients. It helps gauge a patient's ability to perform daily activities and indicates their overall well-being and ability to tolerate treatment.

*Oken, M. M., Creech, R. H., Tormey, D. C., Horton, J., Davis, T. E., McFadden, E. T., & Carbone, P. P. (1982). Toxicity and response criteria of the Eastern Cooperative Oncology Group. American Journal of Clinical Oncology, 5(6), 649-655.*

- 0: Fully active, able to carry on all pre-disease activities without restriction

- 1: Restricted in physically strenuous activity but ambulatory and able to carry out work of a light or sedentary nature

- 2: Ambulatory and capable of all self-care but unable to carry out any work activities; up and about more than 50% of waking hours

- 3: Capable of only limited self-care, confined to bed or chair more than 50% of waking hours

- 4: Completely disabled; cannot perform any self-care; totally confined to bed or chair

- 5: Dead

4. Satava’s Classification: A classification system that categorizes intraoperative complications according to their severity, ranging from Grade 1 (minor) to Grade 5 (major) complications. It helps standardize the reporting of complications during surgical procedures.

*Satava, R. M. (2008). Identification and reduction of surgical error using simulation. Minimally Invasive Therapy & Allied Technologies, 17(4), 319-326.*

- Grade 1: Minor complications

- Grade 2: Serious complications requiring intervention

- Grade 3: Serious complications requiring major intervention

- Grade 4: Life-threatening complications requiring immediate intervention

- Grade 5: Fatal complications

5. Postoperative Bleeding (ISGPS Definition): Bleeding that occurs after surgery, defined according to the International Study Group of Pancreatic Surgery (ISGPS) criteria.

*Bassi, C., Marchegiani, G., Dervenis, C., Sarr, M., Abu Hilal, M., Adham, M., ... & Besselink, M. G. (2017). The 2016 update of the International Study Group (ISGPS) definition and grading of postoperative pancreatic fistula: 11 Years After. Surgery, 161(3), 584-591.*

Grade A: Clinically significant bleeding not requiring transfusion or radiological intervention.

Grade B: Clinically significant bleeding requiring transfusion or radiological intervention.

Grade C: Bleeding requiring surgical re-intervention.

Grade D: Bleeding leading to death.

6. Postoperative Pancreatic Fistula (ISGPS Definition): Leakage of pancreatic fluid after surgery, categorized based on the International Study Group of Pancreatic Surgery (ISGPS) criteria.

*Bassi, C., Marchegiani, G., Dervenis, C., Sarr, M., Abu Hilal, M., Adham, M., ... & Besselink, M. G. (2017). The 2016 update of the International Study Group (ISGPS) definition and grading of postoperative pancreatic fistula: 11 Years After. Surgery, 161(3), 584-591.*

Grade A: Asymptomatic biochemical leak (amylase-rich fluid) with no clinical impact.

Grade B: Clinical impact without requiring specific therapeutic intervention.

Grade C: Clinical impact requiring therapeutic intervention.

Grade C1: Managed without relaparotomy.

Grade C2: Managed with relaparotomy.

7. Postoperative Delayed Gastric Emptying (ISGPS Definition): Delayed resumption of normal gastric emptying after surgery, defined according to the International Study Group of Pancreatic Surgery (ISGPS) criteria.

*Wente, M. N., Bassi, C., Dervenis, C., Fingerhut, A., Gouma, D. J., Izbicki, J. R., ... & Yeo, C. J. (2007). Delayed gastric emptying (DGE) after pancreatic surgery: a suggested definition by the International Study Group of Pancreatic Surgery (ISGPS). Surgery, 142(5), 761-768.*

Grade A: No clinical impact.

Grade B: Delayed gastric emptying requiring therapeutic intervention or prolonging hospital stay.

Grade C: Delayed gastric emptying requiring naso-gastric intubation, enteral nutrition, or total parenteral nutrition.

8. Postoperative Complications Scored and Classified Using Clavien-Dindo Classification: A classification system that categorizes postoperative complications based on their severity, ranging from Grade I (mild) to Grade V (death). It provides a standardized approach for assessing the impact of complications on patient outcomes.

*Dindo, D., Demartines, N., & Clavien, P. A. (2004). Classification of surgical complications: a new proposal with evaluation in a cohort of 6336 patients and results of a survey. Annals of Surgery, 240(2), 205-213.*

- Grade I: Any deviation from the normal postoperative course without the need for pharmacological treatment or surgical, endoscopic, or radiological interventions

- Grade II: Requiring pharmacological treatment with drugs other than those allowed for Grade I complications

- Grade IIIa: Requiring surgical, endoscopic, or radiological intervention

- Grade IIIb: Requiring intensive care unit (ICU) management

- Grade IVa: Life-threatening complication requiring multi-organ failure

- Grade IVb: Single-organ failure (including dialysis)

- Grade V: Death

9. Resection Margins Categorized According to the Royal College of Pathologists Definition: Categorization of the margins (distance between tumor and resection line) based on the definitions provided by the Royal College of Pathologists.

*Royal College of Pathologists. (2001). Minimum dataset for histopathological reporting of pancreatic, ampulla of Vater and bile duct carcinoma. The Royal College of Pathologists.*

- R0: Distance margin to tumor ≥ 1mm (clear margin)

- R1: Distance margin to tumor < 1mm (close margin)

- R2: Macroscopically positive margin
